# LONELY AND SOCIALLY DISCONNECTED OLDER ADULTS ARE AT HIGHER RISK FOR PREVALENT AND INCIDENT DEMENTIA

**DOI:** 10.1093/geroni/igae098.3233

**Published:** 2024-12-31

**Authors:** Carmen Chek, Katherine Knauft, Ariana Stickel, Carlos Araujo-Menendez, Hector Gonzalez, Wassim Tarraf

**Affiliations:** Wayne State University, Detroit, Michigan, United States; Wayne State University, Detroit, Michigan, United States; San Diego State University, San Diego, California, United States; SDSU/UC San Diego, San Diego, California, United States; University of California San Diego, La Jolla, California, United States; Wayne State University, Detroit, Michigan, United States

## Abstract

A 2020 report from the National Academies indicates that nearly one in four adults over age 65 are lonely. Loneliness has important health implications, including for cognitive aging. However, loneliness might not manifest uniformly in the population. Herein, we address a gap in the existing literature by examining the link between heterogeneities in the risk of loneliness and dementia outcomes. We used data from n = 8,805 adults aged 65+ from the Health and Retirement Study (average age 74.7; 56.3% women) to estimate latent classifications of loneliness (11 items from the UCLA loneliness index) and tested, using generalized linear regression models, whether different manifestations of loneliness were differentially associated with dementia (Langa-Weir criteria) prevalence and incidence (over 3-waves of data). A four-class solution best fits the data: “No/Low loneliness” (NL; 51.6%); “Socially disconnected” (SD; 18.0%); “Lonely but not socially disconnected” (LNSD; 12.6%), and “Lonely and socially disconnected” (LSD; 17.8%). Notably, these classifications and their prevalence rates were largely consistent across waves. Adjusting for sociodemographic covariates, compared to individuals in the NL group, the LNSD (OR = 1.68; 95%CI = 1.22;2.32) and LSD (OR = 1.71; 95%CI = 1.33;2.20) groups were more likely to meet criteria for prevalent dementia at baseline. The LSD group was also more likely to meet criteria for incident dementia at follow-ups (within 2 and 4 years from baseline). Loneliness is not uniformly distributed in the population and its subtypes confer differential risk for dementia outcomes. Focusing on the subtypes can potentially pinpoint targetable groups for interventions.

